# Counseling Patients with Chronic Obstructive Pulmonary Disease Traveling to High Altitude

**DOI:** 10.1089/ham.2023.0053

**Published:** 2023-09-12

**Authors:** Konrad E. Bloch, Talant M. Sooronbaev, Silvia Ulrich, Mona Lichtblau, Michael Furian

**Affiliations:** ^1^ Department of Respiratory Medicine, University Hospital Zurich, Zurich, Switzerland.; ^2^ Swiss-Kyrgyz High Altitude Medicine and Research Initiative, Zurich, Switzerland.; ^3^ Swiss-Kyrgyz High Altitude Medicine and Research Initiative, Bishkek, Kyrgyz Republic.; ^4^ Department of Respiratory Medicine, National Center for Cardiology and Internal Medicine, Bishkek, Kyrgyz Republic.

**Keywords:** altitude illness, altitude-related adverse health effects, altitude travel, chronic obstructive pulmonary disease, prevention, mountain sickness

## Abstract

Bloch, Konrad E., Talant M. Sooronbaev, Silvia Ulrich, Mona Lichtblau, and Michael Furian. Clinician's corner: counseling patients with chronic obstructive pulmonary disease traveling to high altitude. *High Alt Med Biol*. 24:158–166, 2023.—Mountain travel is increasingly popular also among patients with chronic obstructive pulmonary disease (COPD), a highly prevalent condition often associated with cardiovascular and systemic manifestations. Recent studies have shown that nonhypercapnic and only mildly hypoxemic lowlanders with moderate to severe airflow obstruction owing to COPD experience dyspnea, exercise limitation, and sleep disturbances when traveling up to 3,100 m. Altitude-related adverse health effects (ARAHE) in patients with COPD include severe hypoxemia, which may be asymptomatic but expose patients to the risk of excessive systemic and pulmonary hypertension, cardiac arrhythmia, and even myocardial or cerebral ischemia. In addition, hypobaric hypoxia may impair postural control, psycho-motor, and cognitive performance in patients with COPD during altitude sojourns. Randomized, placebo-controlled trials have shown that preventive treatment with oxygen at night or with acetazolamide reduces the risk of ARAHE in patients with COPD while preventive dexamethasone treatment improves oxygenation and altitude-induced excessive sleep apnea, and lowers systemic and pulmonary artery pressure. This clinical review provides suggestions for pretravel assessment and preparations and measures during travel that may reduce the risk of ARAHE and contribute to pleasant mountain journeys of patients with COPD.


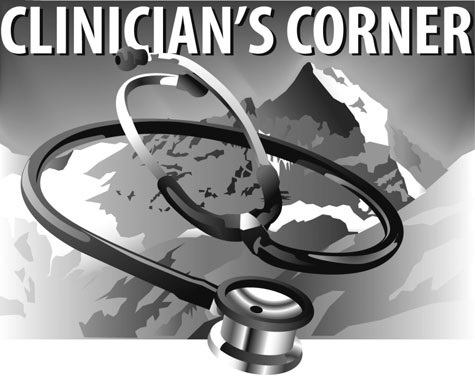


## Introduction

With improving means of transportation and the increasing trend for adventure travel, remote and highly located mountain regions have become easily accessible and favored destinations even for older, physically not very fit tourists, and for patients with preexisting cardiorespiratory disease (World-Tourism-Organisation, 2022). Compared with healthy individuals, patients with preexisting illness may be at increased risk of experiencing unpleasant physical limitations or even altitude-related illness during mountain travel. Until recently, robust scientific data on altitude travel in older persons and in patients with respiratory disease have been very scant. Moreover, almost all previous studies and society recommendations in this field had been focused on preflight assessment and exposure up to an altitude equivalent of ∼2,500 m that corresponds to the minimal cabin pressure allowed during commercial air travel (Bellinghausen and Mandel, 2021; Coker et al., 2022; Nicholson and Sznajder, 2014). Therefore, physicians had to rely on anecdotal evidence when counseling respiratory patients traveling to high altitude.

The purpose of the current clinical review is to summarize recent evidence from research on potential consequences of altitude travel in patients with chronic obstructive pulmonary disease (COPD), one of the most prevalent respiratory diseases (GOLD-Executive-Committee, 2023). In particular, measures that reduce the risk of adverse outcomes will be reviewed as a basis to counsel patients with COPD planning altitude travel. This article complements earlier reviews on altitude travel in patients with the obstructive sleep apnea syndrome (Bloch et al., 2015) and in patients with pulmonary hypertension (Ulrich et al., 2022).

## Chronic Obstructive Pulmonary Disease

COPD is a leading cause of morbidity and mortality worldwide with an estimated prevalence of ∼10% that is expected to rise owing to an increase in cigarette smoking, exposure to biomass combustion, and aging of the population (GOLD-Executive-Committee, 2023). Accordingly, the prevalence of COPD is also expected to rise among altitude travelers. The main causes of COPD include smoking and inhalation of irritant and toxic substances, but in a considerable portion of COPD patients no known risk factors can be identified. The diagnosis of COPD relies on the typical symptoms of dyspnea, exercise limitation, chronic cough, and exacerbations in the presence of nonfully reversible airflow obstruction. According to the Global Initiative for Chronic Obstructive Pulmonary Disease (GOLD), spirometric criteria that confirm the diagnosis of COPD are a postbronchodilator ratio of the forced expiratory volume in 1 second (FEV1) to the forced expiratory vital capacity of <0.7 (GOLD-Executive-Committee, 2023).

Based on the value of the FEV1 in percent of the predicted value, the severity of airflow limitation is graded as mild (FEV1 ≥ 80%pred., GOLD 1), moderate (FEV1 50%–79%pred., GOLD 2), severe (FEV1 30%–49%pred., GOLD 3), and very severe (FEV1 < 30%pred., GOLD 4). The symptoms and risk of exacerbations are further represented by the GOLD ABE assessment tool. The main treatment options for COPD include avoidance of noxious exposures, in particular, smoking cessation, inhaled bronchodilators (long-acting beta-adrenergics and anticholinergics), and in selected cases inhaled corticosteroids and supplemental oxygen. Vaccinations for respiratory pathogens, physical activity, and appropriate nutrition are important adjunctive measures. Exacerbations are treated with corticosteroids, antibiotics, supplemental oxygen, and mechanical ventilatory support in those with hypercapnic respiratory failure. In very severe cases, lung volume reduction and lung transplantation are further treatment options.

## Effect of Altitude in COPD patients

Patients with COPD are predisposed to adverse effects of altitude for several reasons: their ability to augment ventilation in response to hypoxemia may be limited by airflow obstruction, hyperinflation, and a reduced strength of the respiratory muscles. The pulmonary gas exchange of patients with COPD may be impaired by ventilation/perfusion mismatch (Neder et al., 2022) and loss of alveolar-capillary surface owing to pulmonary emphysema, and their pulmonary circulation may not adequately adapt to the increased demand for blood flow in hypoxic conditions (O'Donnell et al., 2017). Moreover, some patients with severe COPD may have a disturbed control of breathing during wakefulness and sleep predisposing to hypoventilation and sleep apnea (Latshang et al., 2019).

As a systemic disease that affects older persons and several organ systems other than just the lungs, COPD is often accompanied by comorbidities (GOLD-Executive-Committee, 2023), in particular, cardiovascular disease, such as coronary heart disease and cerebrovascular disease, which increase the risk of myocardial infarction and stroke (Rothnie et al., 2016). Hypoxia-induced impairments in postural control (Muralt et al., 2018) and in cognitive (Kourtidou-Papadeli et al., 2008) and visuomotor performance (Scheiwiller et al., 2022) represent additional risks and hamper activities of patients with COPD during altitude sojourns.

In a survey among commercial airplane passengers, patients with COPD more commonly reported in-flight symptoms of dyspnea and air hunger than passengers without respiratory disease (Edvardsen et al., 2011). Accordingly, studies in patients with COPD have revealed a more severe altitude-induced reduction in arterial oxygen saturation compared with healthy individuals of similar age (Christensen et al., 2000; Furian et al., 2022b), a reduction in exercise capacity (Furian et al., 2018b; Kelly et al., 2009), and a propensity to excessive altitude-induced sleep apnea (Latshang et al., 2019) as well as a rise in systemic and pulmonary artery blood pressure with impairments in left and right heart function (Lichtblau et al., 2019b) during high-altitude exposure.

## Altitude-Related Adverse Health Effects in COPD

Previous studies and clinical guidelines on prevention and treatment of altitude illness have focused on specific, symptomatic diseases such as acute mountain sickness (AMS), high-altitude cerebral edema (HACE), and high-altitude pulmonary edema (HAPE) in young, healthy altitude travelers (Luks et al., 2019). Conversely, asymptomatic altitude-induced hypoxemia or hypoxemia merely associated with dyspnea or exercise limitation has been of little concern. This attitude appears not appropriate for people of older age who are at risk of having unrecognized comorbidities and for those with already known preexisting respiratory or cardiovascular disease. Indeed, clinical experience indicates that older persons with COPD, in particular those with cardiovascular comorbidities, might be at risk of complications when exposed to hypoxemia as it enhances ventilatory drive which, in the presence of airflow obstruction and limited ventilatory reserve, may exacerbate dyspnea, worsen dynamic hyperinflation, and result in overexcitation of sympathetic tone with associated adverse cardiovascular and systemic consequences including myocardial and cerebral ischemia (Bisang et al., 2021; Meszaros et al., 2021; O'Donnell et al., 2017).

Thus, hypoxemia *per se*, as well as manifestations of cardiorespiratory and other comorbidities may be clinically as important for patients with COPD traveling to high altitude as the acute altitude illnesses mentioned previously. Therefore, in the presented studies in patients with COPD, a comprehensive endpoint termed altitude-related adverse health effects (ARAHE, defined in Table 1) was used to capture any condition that may be potentially harmful and impair well-being of patients during altitude travel.

**Table 1.** Altitude-Related Adverse Health Effects

Severe hypoxemia	Pulse oximetry (Spo_2_) at rest <80% for >30 minutes, or <75% for >15 minutes
Symptomatic cardiovascular disease	Arterial hypertension, systolic >200 mmHg, diastolic >110 mmHg, not responding to blood pressure lowering drugs within 1 hour Chest pain with ECG signs of ischemia or new onset arrhythmia
Acute mountain sickness	LLS ≥3 and/or Environmental Symptoms cerebral score (AMSc) ≥0.7
Subjective discomfort	Desire of the person to descend to lower altitude or to receive oxygen or other medical treatment because of any symptoms or health concerns
Medical concern	Need of oxygen supplementation, medical treatment or transport to lower altitude because of symptoms or health concerns according to the decision of an independent physician

This term has been used as an endpoint in various clinical studies to capture any condition that may impair the well-being or safety of altitude travelers.

AMSc, Environmental Symptoms Questionnaire cerebral subscore evaluating acute mountain sickness; LLS, Lake Louise score.

## Studies on Prevention and Treatment of ARAHE in COPD Patients

According to clinical reasoning, patients with COPD with very severe airflow obstruction, hypoxemia, or hypercapnia already near sea level should not travel to high altitude without using supplemental oxygen as they are at high risk of adverse outcome. Therefore, the studies discussed below have excluded these severely ill patients for safety and ethical reasons.

An important question that COPD patients going to high altitude want to know is whether they might benefit from using supplemental oxygen. To address this point, we performed a randomized, placebo-controlled crossover trial in normocapnic, not-severely hypoxemic lowlanders with moderate to severe COPD (pulse oximetry ≥92%, FEV1 30%–80% of predicted, near sea level) (Tan et al., 2020). Patients were studied at 2,050 m twice, for 2 days and nights each time, in randomized order with a washout period of at least 2 weeks in-between. During one stay, they used nocturnal oxygen (3 l/min per nasal cannula, i.e., the maximal rate delivered by current portable oxygen concentrators); during the other stay, they used sham oxygen (room air). The coprimary outcomes, the mean nocturnal oxygen saturation, and the apnea/hypopnea index were significantly improved with nocturnal oxygen compared with sham oxygen.

In addition, the number of arousals from sleep and sleep efficiency were improved with oxygen. Even more importantly, nocturnal oxygen reduced the incidence of ARAHE from 26% to 4% (odds ratio = 0.10; 95% confidence interval, 0.01–0.88, *p* = 0.04) in these patients (Tan et al., 2020).

Because oxygen administration is often not feasible during altitude travel, we evaluated other, more convenient medical treatments for prevention of ARAHE in patients with COPD. Glucocorticoids are used to treat COPD exacerbations (GOLD-Executive-Committee, 2023), and dexamethasone, a drug with potent glucocorticoid action, is used for prevention and treatment of AMS and HACE in healthy individuals (Nieto Estrada et al., 2017). Moreover, dexamethasone reduces pulmonary artery pressure and enhances ventilation in HAPE-susceptible persons at high altitude (Maggiorini et al., 2006). The mechanisms of action of dexamethasone in this setting are still incompletely understood but modulation of sympathetic tone, systemic inflammation, vascular endothelial function, alveolar fluid clearance, and ventilatory control have all been proposed to play a role (Swenson, 2016). Therefore, we reasoned that dexamethasone treatment might prevent ARAHE in patients with mild to moderate COPD.

However, in a randomized, double-blind, parallel design trial, dexamethasone (one 4 mg tablet twice daily) did not reduce the incidence of ARAHE compared with placebo (Furian et al., 2018c). Nevertheless, dexamethasone mitigated the altitude-induced systemic and cerebral hypoxemia (Furian et al., 2021) and sleep apnea (Furian et al., 2019) and the rise in systemic and pulmonary artery pressure (Lichtblau et al., 2019a) in patients with COPD.

**FIG. 1.**
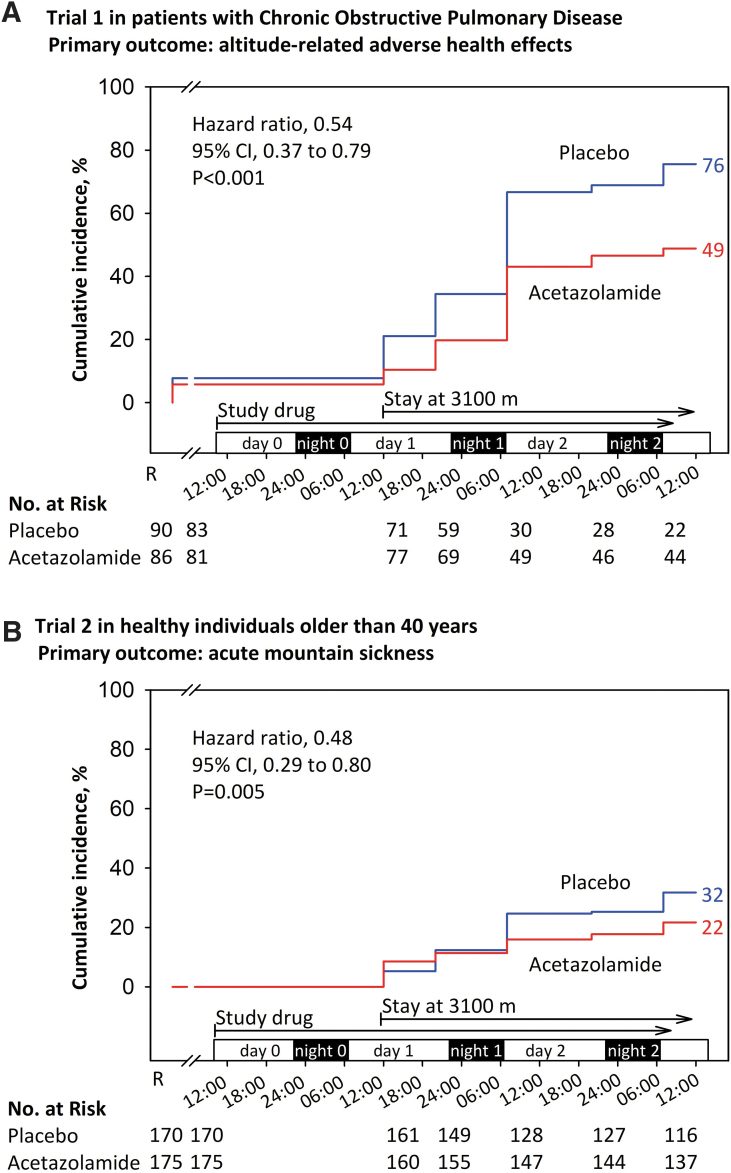
Kaplan–Meier curves representing the results of two randomized, placebo-controlled, double-blind trials evaluating efficacy of preventive acetazolamide therapy in reducing the incidence of ARAHE in patients with **(**COPD, **A)** and AMS in healthy individuals, 40 years of age or older **(B)**. The y-axis represents the cumulative incidence of the primary outcome ARAHE and AMS, respectively. After baseline evaluation at 760 m and randomization (R), participants started taking the study drug (either acetazolamide, 375 mg/day or placebo) on day 0, around noon. On day 1, in the morning, participants traveled by minibus to 3,100 m and arrived there in the afternoon. Night rest was from around 22:00 to 06:00 hours (*black boxes*). *Blue lines* denote data from the placebo group and *red lines* data from the acetazolamide group. Chi-square statistics indicated a reduced incidence of ARAHE in patients with COPD taking acetazolamide versus those taking placebo. In healthy older individuals, acetazolamide reduced the incidence of AMS compared with placebo. AMS, acute mountain sickness; ARAHE, altitude-related adverse health effects; CI, confidence interval; COPD, chronic obstructive pulmonary disease. Reproduced from Furian et al. (2022b) NEJM Evidence 2022.

The carbonic anhydrase inhibitor acetazolamide is widely used for prevention and treatment of AMS and HACE (Luks et al., 2019). Its beneficial effects on oxygenation and AMS have been mainly ascribed to the stimulation of ventilation by lowering the arterial pH through enhanced urinary bicarbonate excretion. Carbonic anhydrase inhibition in the central nervous system and in vascular endothelial cells may additionally account for the clinical effects of acetazolamide (Swenson, 2016). In outpatients with hypercapnic COPD, acetazolamide has been used to enhance oxygenation (Adamson and Swenson, 2017). To evaluate the efficacy of acetazolamide in prevention of ARAHE in patients with COPD, we performed a randomized, placebo-controlled, double-blind, parallel design trial in 176 lowlanders with COPD traveling to and staying for 2 days at 3,100 m. We found a significant reduction in the incidence of ARAHE (Fig. 1) associated with an improved oxygenation (Furian et al., 2022b).

The number of patients needed to treat to prevent one case of ARAHE was 4. The drug was well tolerated, no serious adverse effects occurred. In a related trial in 345 healthy individuals of similar age as the patients with COPD, that is, 40 years or older, acetazolamide significantly reduced the incidence of AMS and ARAHE (Furian et al., 2022b). The dose of acetazolamide in these studies was 125 mg in the morning and 250 mg in the evening based on the assumption that a higher evening dose would mitigate sleep-related hypoventilation, whereas the lower dose in the morning would not excessively stimulate ventilation so that triggering dyspnea could be avoided.

These studies also demonstrated that acetazolamide improved daytime and nocturnal oxygenation, high-altitude periodic breathing, and attenuated the altitude-induced elevation of systemic and estimated pulmonary blood pressure (Furian et al., 2022b; Lichtblau et al., 2023). Moreover, preventive acetazolamide treatment improved altitude-induced impairment in visuomotor performance and overnight learning both in patients with COPD (Scheiwiller et al., 2022) and healthy, older individuals (Reiser et al., 2023).

## Clinical Recommendations to COPD Patients Traveling to High Altitude

Pretravel evaluations and timely preparations as well as measures to be taken during an altitude sojourn in the event of ARAHE are intended to assure a pleasant and safe altitude travel of patients with COPD. Corresponding recommendations are summarized in Tables 2 and 3.

**Table 2.** Counseling Patients with Chronic Obstructive Pulmonary Disease Planning Altitude Travel

Pretravel evaluation	
History	Medical history with particular focus on respiratory disease, cardiovascular disease, sleep apnea, diabetes, and any other comorbidities Functional class, physical fitness Stability of the patient's condition (exacerbations, respiratory infections) Established treatment: inhaled bronchodilators, other medication Additional medical treatment Dependency on electrical power (for inhalations, other devices), oxygen concentrator, ventilatory support (CPAP, BiPAP devices) Previous altitude sojourns and tolerance? Maximum altitude reached, with/without overnight stay? Tolerance of physical exertion?
Physical examination and complementary evaluations	Vital signs, signs of right heart failure, pulse oximetry Spirometry Arterial blood gas analysis and diffusing capacity measurement if resting pulse oximetry near sea level is <92% Depending on specific setting: 6-minute walk test with pulse oximetry to obtain an estimate of general fitness and physical impairment at altitude Additional evaluations such as, cardiopulmonary exercise test, echocardiography according to clinical judgment (see text)
Travel plans in detail	Maximal altitude to be reached, ascent rate, duration of sojourn, with/without overnight stay Setting, accessibility and facilities at destination: transportation (car, public transportation, plane, cable car), electrical power, medical facilities Requirement and degree of physical exertion Accompanying persons Requirement of regular and additional medication Depending on the travel setting (duration, opportunity to descend rapidly) and individual patient (experience from previous altitude travel), preventive treatment with acetazolamide might be considered during the altitude sojourn. If this is planned, a treatment trial at low altitude for 2 days is suggested (oral acetazolamide tablets 125 mg, one in the morning, two in the evening).
Conclusion on altitude sojourn	Feasible as planned Too risky, should be discouraged. This applies particularly to unstable patients with intercurrent illness, COPD exacerbation within the last month, unstable cardiovascular or neurological disease Further measures required, postpone, use oxygen during travel Evaluation by a pulmonary specialist with experience in altitude medicine Adaptation of route, destination, logistics (other means of transportation, lower altitude, no overnight stay, supplemental oxygen, etc.) Reevaluation after optimization of condition/treatment.

Pretravel preparations	
Logistics	Type of transportation Choose facility at destination for resting, food, electricity, overnight stay as appropriate Get information on medical facility, emergency service Travel in company of a person who knows about your condition Consider pulse oximetry monitoring (spot check) Consider taking a validated blood pressure measuring device
Treatment	Prepare all medication, including emergency medication and additional supply in case of unexpected delay of trip Arrange for supplemental oxygen in case of emergency as appropriate Decide on preventive treatment with nocturnal oxygen during the stay at altitude and/or acetazolamide starting 24 hours before ascent and during the stay at high altitude Measures to alleviate discomfort from nasal dryness and assure nasal patency should be prepared (see text).
Emergency	Have checklist for emergency self-treatment Take along current medical report with all relevant information Contact details on relevant services (transportation, medical facilities).

BiPAP, bilevel positive airway pressure; COPD, chronic obstructive pulmonary disease; CPAP, continuous positive airway pressure.

**Table 3.** Recommendations to Chronic Obstructive Pulmonary Disease Patients During High Altitude Travel

Activities	Avoid strenuous physical activities, be flexible to allow enough time for brakes and rest as needed
Preventive acetazolamide and/or oxygen treatment	According to pretravel advice by the pulmonologist, nonhypercapnic and not-severely hypoxemic patients with mild-to-moderate airflow obstruction at low altitude (FEV1 > 40% predicted, Spo_2_ ≥92%, PaCO_2_ ≤6.0 kPa) may start preventive acetazolamide treatment 1 day before ascent and during the stay at altitude (acetazolamide tablets 125 mg, one in the morning, two in the evening) According to pretravel advice and feasibility, preventive nocturnal oxygen supplementation at 2–3 l/min per nasal cannula may be used
General measures in case of ARAHE	In case of impaired subjective well-being, physical activity should be reduced and a period of rest should be maintained as feasible and appropriate. Inhaled bronchodilators may provide relief of dyspnea Complementary monitoring of pulse oximetry and blood pressure may help to identify a condition that requires medical intervention Depending on the setting and feasibility, descent to lower altitude and/or treatment with supplemental oxygen should be considered and initiated
Symptoms and/or signs of impending ARAHE	In case of subjective unwellness, excessive dyspnea and/or other symptoms, pulse oximetry may be checked and further measures may be initiated and prioritized according to the result. Start with supplemental oxygen treatment Nonhypercapnic patients with mild to moderate airflow obstruction might consider starting acetazolamide (tablets 125 mg, starting dose 2, maintenance dose 2 in the evening, one in the morning)
COPD exacerbation and/or respiratory infection	Consider starting glucocorticoids, antibiotics and additional bronchodilators according to pretravel prescription by the general physician or pulmonologist
Cardiovascular decompensation	Consider taking antihypertensive drugs, diuretics, nitroglycerine and/or other drugs according to pretravel prescription by the general physician or pulmonologist/cardiologist Assess the need for and the urgency of evacuation to lower altitude as well as the available and suitable means of transportation

ARAHE, altitude-related adverse health effects; FEV1, forced expiratory volume in 1 second.

During pretravel evaluation, a thorough medical history and clinical evaluation are obtained in conjunction with spirometry and pulse oximetry. If pulse oximetry shows a value <92%, arterial blood gas analysis may help to better assess the need for oxygen during altitude travel based on values of the arterial oxygen partial pressure (PaO_2_) and the alveolar–arterial oxygen partial pressure gradient (PaaO_2_); moreover, arterial blood gas analysis may reveal hypercapnic respiratory failure in patients with severe COPD or suggest the presence of sleep-related hypoventilation by (posthypercapnic) metabolic alkalosis (Berend et al., 2014).

Studies in patients with COPD have shown that the altitude-induced reduction in the 6-minute walk distance or in the endurance time during submaximal constant load cycling exercise is correlated with low-altitude baseline performance (Furian et al., 2018a; Gutweniger et al., 2021). Therefore, exercise tests may help to better assess the expected physical performance decrement of a patient with COPD during altitude travel. Moreover, a formal cardiopulmonary exercise test may quantify physical fitness and indicate the presence of coronary heart disease, poorly controlled hypertension, and other relevant cardiopulmonary diseases. In patients with suspected heart disease, evaluations may be supplemented by an echocardiography, although the potential role of echocardiography in pre-altitude travel evaluation has not been studied.

The role of a hypoxia altitude simulation test in predicting the risk of ARAHE in COPD patients is limited because of a low negative predictive value (Bauer et al., 2023). Based on the described evaluation and the specific travel plans, patients are counseled against or in favor of undertaking the trip while following further recommendations. Patients and accompanying persons may be involved in the assessment and decide on any appropriate adaptation of travel plans.

The individual, specific recommendations are based on clinical judgment, general treatment guidelines for COPD according to GOLD (GOLD-Executive-Committee, 2023), and on the recent randomized trials discussed previously. On this basis, hypoxemic patients with severe COPD (GOLD grades 3–4) fulfilling criteria for oxygen therapy at low altitude should travel to high altitude only with adequate oxygen supplementation. Hypercapnic patients on long-term noninvasive ventilation with or without supplemental oxygen therapy should use their ventilator and oxygen supplementation at appropriate settings to maintain a target oxygen saturation of 88%–92% during altitude travel (Schwarz and Bloch, 2019). Of importance, they should carry along all necessary equipment to assure uninterrupted respiratory support. If these prerequisites are fulfilled and in an appropriate setting in terms of means of transportation, availability of medical support, and opportunity to descend to lower altitude, we feel that altitude travel may still be feasible and safe for patients with severe COPD.

As a rule, the patient should be in stable condition for at least 2 weeks before undergoing altitude travel and should have recovered from any COPD exacerbation for at least 1 month as the risk of cardiovascular events is considerably elevated during this period (Dransfield et al., 2022). The usual medication should be continued during altitude travel. Taking along sufficient supply of these drugs as well as of emergency medication for COPD exacerbations, respiratory infections, and acute altitude illness is essential.

Because of the dry and cold air and the increased ventilation at high altitude, nasal obstruction is common. It is not only unpleasant but may trigger dyspnea and hamper effective nasal oxygen administration. Therefore, patients prone to nasal obstruction are advised to use a humidifying nasal cream, apply nasal saline irrigations in case of crusted secretions, and have a decongestant nasal spray ready for short-term application during altitude travel.

Structured self-monitoring by a symptom checklist and pulse oximetry may help to identify patients with impending ARAHE at an early stage (Furian et al., 2022a). According to preliminary data from the cited study, patients with COPD may check their oxygen saturation by a pulse oximeter at regular intervals during altitude travel, that is, three times over the course of a day, and self-assess any symptoms of AMS. If stable resting pulse oximetry readings are <85% and/or if more than mild symptoms of AMS emerge, the patient is at high risk of impending ARAHE and recommended to start using oxygen (if available) or descend to lower altitude.

If logistically feasible, using supplemental oxygen during physical exertion and/or during sleep at high altitude is beneficial for most normocapnic COPD patients with moderate to severe airflow obstruction (Tan et al., 2020). It is particularly recommended to those who fulfill criteria for long-term oxygen therapy (i.e., resting PaO_2_ <7.3 kPa near sea level). We suggest that oxygen concentrators are operated in continuous rather than pulsed flow mode to account for the risk of trigger failure owing to nasal obstruction and oral breathing route at altitude (Akero et al., 2011). Battery-driven mobile concentrators capable of delivering 2–3 l/min continuous flow oxygen are available and have been successfully used by us at altitudes up to 3,200 m (e.g., the SimplyGo; Philips Respironics, Murrysville, or the Eclipse 5; Caire, Inc., Ball Ground).

Normocapnic and not severely hypoxemic patients with mild to moderate COPD (FEV1 > 40% predicted) at low altitude may benefit from preventive treatment with acetazolamide at a dose of 125 mg in the morning and 250 mg in the evening starting 24 hours before ascent and continued during the stay at altitude (Furian et al., 2022b). This preventive treatment may be particularly useful during prolonged travel without opportunity to use supplemental oxygen or to descend rapidly.

In the event of an intercurrent illness such as COPD exacerbation, the same treatment as at low altitude should be initiated. Oxygen therapy and a timely descent to lower altitude should be considered and, if deemed appropriate, performed as soon as logistically feasible. In case of a COPD exacerbation, dexamethasone (4 mg tablets twice per day) may be used as systemic glucocorticoid both to reduce inflammation and improve oxygenation (Furian et al., 2019).

## Conclusions

Clinicians have to be aware that patients with impaired lung function and comorbidities such as those with COPD require specific counseling when planning altitude travel. Compared with young, healthy individuals, older patients with COPD may experience more pronounced dyspnea and limitations in physical performance owing to the combined effects of impairments in ventilation and gas exchange and because of cardiovascular comorbidities. In addition to ARAHE, disturbances in sleep, postural control and psychomotor function may impair the well-being and safety of patients with COPD during altitude sojourns. Individual recommendations for altitude travel in patients with COPD have to account for the severity of airflow obstruction and gas exchange impairment and the setting of the planned travel. Preventive measures start already during pretravel preparations and include detailed information and advice on measures in case of an impending or overt ARAHE.

Although recent research has provided important information on physiological and clinical effects of high-altitude exposure, further studies are needed to corroborate and refine recommendations for altitude travel in patients with COPD.
